# Responsiveness to PI3K and MEK Inhibitors in Breast Cancer. Use of a 3D Culture System to Study Pathways Related to Hormone Independence in Mice

**DOI:** 10.1371/journal.pone.0010786

**Published:** 2010-05-26

**Authors:** Maria Laura Polo, Maria Victoria Arnoni, Marina Riggio, Victoria Wargon, Claudia Lanari, Virginia Novaro

**Affiliations:** Laboratory of Hormonal Carcinogenesis, Institute of Experimental Biology and Medicine (IBYME)-National Council for Scientific and Technical Research (CONICET), Buenos Aires, Argentina; City of Hope National Medical Center, United States of America

## Abstract

**Background:**

A significant proportion of breast cancer patients face failure of endocrine therapy due to the acquisition of endocrine resistance. We have explored mechanisms involved in such disease progression by using a mouse breast cancer model that is induced by medroxyprogesterone acetate (MPA). These tumors transit through different stages of hormone sensitivity. However, when cells from tumor variants were seeded on plastic, all were stimulated by progestins and inhibited by antiprogestins such as RU486. Furthermore, cells from a RU486-resistant tumor variant recovered antiprogestin sensitivity.

**Hypothesis:**

A three-dimensional (3D) culture system, by maintaining differential cellular organization that is typical of each tumor variant, may allow for the maintenance of particular hormone responses and thus be appropriate for the study of the effects of specific inhibitors of signaling pathways associated with disease progression.

**Method:**

We compared the behavior of tumors growing *in vivo* and cancer cells *ex vivo* (in 3D Matrigel). In this system, we evaluated the effects of kinase inhibitors and hormone antagonists on tumor growth.

**Principal Findings:**

LY294002, a PI3K/AKT pathway inhibitor, decreased both tumor growth *in vivo* and cell survival in Matrigel in MPA-independent tumors with higher AKT activity. Induction of cell death by anti-hormones such as ICI182780 and ZK230211 was more effective in MPA-dependent tumors with lower AKT activity. Inhibition of MEK with PD98059 did not affect tumor growth in any tested variant. Finally, while Matrigel reproduced differential responsiveness of MPA-dependent and -independent breast cancer cells, it was not sufficient to preserve antiprogestin resistance of RU486-resistant tumors.

**Conclusion:**

We demonstrated that the PI3K/AKT pathway is relevant for MPA-independent tumor growth. Three-dimensional cultures were useful to test the effects of kinase inhibitors on breast cancer growth and highlight the need for *in vivo* models to validate experimental tools used for selective therapeutic targeting.

## Introduction

### Signaling pathways in breast tumor progression

About two-thirds of breast cancers express a functional estrogen receptor (ER) and are initially dependent on 17β-estradiol for growth and survival. However, eventually some of these cancers progress to hormone independence [1]. Endocrine therapies, which inhibit ER signaling, are the most common and effective treatments for ERα-positive breast cancer. These include the selective ER down-regulators tamoxifen and fulvestrant (ICI182780) [2] and the aromatase inhibitors [3]. However, the use of these agents is limited by the frequent development of resistance after prolonged treatment. Another steroid receptor that has gained special attention in the last years of research on breast cancer is the progesterone receptor (PR). Endocrine therapies using mifepristone (RU486) [4], [5] or ZK230211 [6], [7] that block the function of PR have not yet been extended into patients and more preclinical studies are required to understand their mechanisms of action.

Several studies have focused on the compensatory cross-talk between steroid receptors and various signaling pathways activated by tyrosine kinases associated with growth factor receptors [1], [8], [9]. These studies have shown that such cross-talk may account for the autonomous growth and for the progression to decreased sensitivity to steroid receptor antagonists in breast cancer. In particular, activation of the phosphatidylinositol-3-OH kinase (PI3K)/Protein kinase B (AKT/PKB) survival pathway has been implicated in the progression of endocrine-resistant tumors [10]–[12] and has been associated with poor prognosis [13], [14]. The same studies suggest that AKT is a potential target for the development of new antitumor therapies. Another kinase that is involved in the progression of hormone resistance is mitogen-activated protein kinase (MAPK)/extracellular signal-regulated kinase (ERK) [15], and specific inhibitors of ERK kinase (MEK) have been developed that efficiently inhibit the oncogenic RAS-MEK-ERK pathway.

During the translation of basic science, it is still inevitable that some of the treatments do not work, or after a variable period of time under treatment, refractory mechanisms arise and tumor relapse occurs [1], [15]. One reason for the relapse might stem, as mentioned above, from alterations in the activity of signaling pathways in a given tumor. Another reason is the variability in the behavior among different tumor variants, which results from the intrinsic heterogeneity of tumor cells (genetic and epigenetic) [16] and the heterogeneous environment in which the cells reside inside the tumor [17]–[19]. Hence, cancer therapy agents that induce apoptosis can be effective for some kinds of tumors but not for others. For these reasons, understanding the sources of this variability might have a significant therapeutic impact.

### Tumor microenvironment

All components of the mammary gland, in addition to the luminal and/or tumor epithelial cells, are instrumental in maintaining organ integrity and promoting and, at times, even initiating breast cancer development [18], [20]. Consequently, important signals are lost when cells are cultured *ex vivo* on two-dimensional (2D) plastic substrata. Many of these crucial microenvironmental cues may be restored by generating three-dimensional (3D) cultures that use laminin-rich extracellular matrix (commercial Matrigel). This model provides an excellent system to study tissue organization, epithelial morphogenesis [21], [22], and breast carcinogenesis [23]–[25] in a more physiological context.

Paradigmatic studies in Dr. Bissell's laboratory have shown that it is possible to revert the malignant phenotype by targeting environmental factors [24], [26], [27] and by correcting alterations in signal transduction pathways [28], [29], both *in vivo* and in culture, without altering the genetic lesions of the tumor, summarized in [30], [31].

### Mouse mammary tumor model

The number of relevant and well-characterized animal models for studying breast cancer is small [32]–[34], and this represents a limitation for research in the field. With the aim of developing new experimental systems for *in vivo* studies of hormone-dependent and -independent tumor growth, progression and invasion, we have made use of a murine experimental model of breast cancer that is induced by the progesterone analog medroxyprogesterone acetate (MPA) [35], [36]. The original tumor variant requires the administration of MPA to grow (hormone-dependent, HD). Spontaneously, a group of tumors begin to grow in the absence of MPA (hormone-independent, HI) [37]. These two tumor variants retain a ductal phenotype and maintain functional ER and PR [37], [38] reviewed in [39]. However, a member of HI tumors, C4-HI, display a more differentiated pattern [7], compared to a member of HD tumors, C4-HD [40]. Therefore, as is often found in the clinic, loss of hormone dependence in this model was not due to the loss of expression of steroid receptors. Moreover, a recent study reported that carcinoma-associated fibroblasts derived from C4-HI tumors produce higher levels of fibroblast growth factor-2 (FGF-2) than fibroblasts derived from C4-HD tumors [41]. Whereas C4-HD and C4-HI tumors regress after treatment with RU486 or tamoxifen [40], [42], another tumor variant with acquired resistance to antiprogestin therapy, C4-HIR, was obtained by prolonged selective pressure of C4-HI tumors with RU486. This variant exhibits greater activation of ERK and metastatic potential [7].

Thus, the MPA model progresses through different stages of hormone responsiveness [7], [40], [42], and it is especially useful for studies of hormone receptor function, protein kinase involvement and the role of stromal elements in tumor progression. Together, the evidence suggests that changes in the signaling pathways involving steroid receptor regulation, rather than loss of expression, might impact tumor susceptibility to treatment. However, the signaling pathways involved in the different tumor phenotypes are still unidentified in the MPA model.

In this study, the 3D Matrigel culture system, by preserving the physiologically relevant microenvironment that more closely mimics tumor architecture, causes cancer cells to function as they do *in vivo*. In this system, we show that AKT activation is involved in ERα expression and in the progression of MPA-induced mammary tumors to a hormone-independent phenotype. Furthermore, we proved our hypothesis that the activation of particular signaling pathways depends on the interaction of epithelial tumor cells with their microenvironment. However, the 3D Matrigel system is still insufficient to reproduce the responsiveness of acquired tumor resistance. The ultimate goal is to use this model to develop a preclinical assay to predict cancer sensitivity to antitumor agents in order to prevent or delay the surge of hormone-independent and endocrine-resistant tumor variants.

## Results

### PI3K/AKT signaling pathway regulates growth of C4-HI but not C4-HD tumors

In order to understand the mechanisms involved in the transition from hormone-dependent to hormone-independent mammary tumors, we have focused our study on the role of PI3K and of MEK induced signaling, as deduced by assessment of AKT and ERK1/2 phosphorylation after exposure to PI3K and MEK inhibitors, respectively.

**Figure 1:**
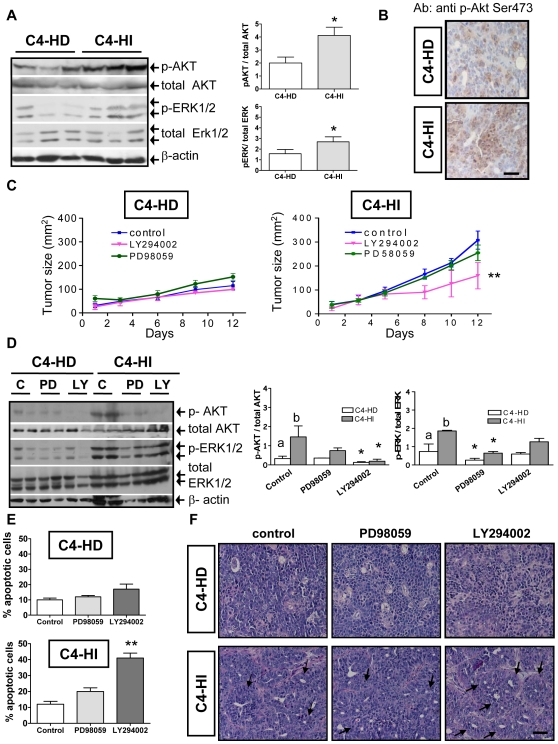
The PI3K/AKT pathway regulates growth in C4-HI but not in C4-HD tumors. C4-HD tumors were transplanted into BALB/c females carrying a MPA depot while C4-HI tumors were transplanted in the absence of MPA. When tumors reached a 150 mm^2^ of size, they were removed and processed as described in Materials and Methods for western blot or immunohistochemistry. **A**. Left. Western blots of protein extracts from C4-HD and C4-HI tumors using specific antibodies against phosphorylated Ser473 AKT (p-AKT), total AKT, phosphorylated ERK1/2 (p-ERK1/2), total ERK1/2 and β-actin (loading control). Three representative samples of a total of six of each tumor type are shown. Right. Quantification of AKT activation (p-AKT relative to total AKT levels) and ERK activation (p-ERK relative to total ERK levels). n = 6 for each tumor type; *: p<0.05 C4-HI vs. C4-HD. **B**. Immunohistochemistry of slices of paraffin embedded C4-HD or C4-HI tumors using antibodies against phosphorylated Ser473 AKT, showing a higher number of p-AKT-positive cells in C4-HI than in C4-HD tumors. Brown corresponds to the antibody signal and blue marks nuclei stained with hematoxylin. Scale bar: 60 µm. **C**. 3.6 mg/kg PD98059 (MEK inhibitor), 4 mg/kg LY294002 (PI3K inhibitor) or 100 µl of saline solution (c, control) were administrated i.p. every other day for 12 days to animals carrying C4-HD or C4-HI tumors. Length and width of mammary tumors (mm^2^) were measured every 2 days. Treatments with the inhibitors started once the tumors reached a size of approximately 30 mm^2^. Treatment with LY294002 causes C4-HI tumors to have a decreased growth rate. n = 6 for each group; **: p<0.01 vs. control. **D**. Left. At the end of 12 days of treatment, tumors were removed to evaluate by western blot the inhibitory effect of PD98059 and LY294002 on p-ERK1/2 and p-AKT, respectively. Two representative samples of a total of six are shown in the gel (left). Right. Quantification of AKT and ERK activation as in A. n = 6 for each group; a,b: p<0.01 C4-HI vs. C4-HD; *:p<0.05 vs. control. **E**. Quantification of the percentage of apoptosis at the end of treatment. Data corresponds to the mean±SEM percentage of apoptotic cells in ten high-power fields corresponding to six independent experiments. Apoptosis is higher in C4-HI tumors treated with LY294002. **: p<0.01 vs. control. **F**. C4-HI tumors are more differentiated than C4-HD tumors. After 12 days of treatment with LY294002, there is an increase in the number of ductal-like structures only in the C4-HI tumors, as indicated by the black arrows. Scale bar: 30 µm.

Analysis by western blotting revealed that, in comparison with C4-HD tumors, C4-HI tumors exhibit higher activation of both AKT and ERK1/2 (p<0.05; Figure 1A). Kinase activation level was quantified as the ratio of phosphorylated Ser473 AKT (p-AKT) to total AKT, and the ratio of phosphorylated ERK1/2 (p-ERK1/2) to total ERK1/2, respectively (Figure 1A, bar graphs). Immunohistochemistry analysis showed a more intense signal for p-AKT in C4-HI tumors (Figure 1B), confirming western blots results.

The involvement of the two signaling pathways in mammary tumor growth was evaluated using specific inhibitors: PD98059, an inhibitor of MEK1, and LY294002, an inhibitor of PI3K. Briefly, 3.6 mg/kg PD98059, 4 mg/kg LY294002 or 100 µl of saline solution (control) were administrated i.p. to animals carrying C4-HD or C4-HI tumors as indicated in Materials and Methods.

Neither of the inhibitors could interfere with C4-HD tumor growth (Figure 1 C left). In contrast, a significant decrease in tumor growth was observed in C4-HI tumors treated with LY294002 (p<0.01; Figure 1C right), indicating that the activity of the PI3K/AKT pathway is necessary for C4-HI tumors to grow. Similar results were found in C4-HI tumors growing in the presence of MPA (not shown), indicating that the differential effect of LY294002 in the two tumor variants was not due to the influence of the progesterone analog.

It is important to point out that the growth rate of C4-HI tumors growing with (not shown) or without MPA (Figure 1C) was higher than the rate of C4-HD tumors growing with MPA. This is not surprising since we have already reported that the growth rate depends on the number of passages used in each tumor line, and C4-HI tumors include more passages than the original C4-HD tumors [39].

Even though the activation of ERK1/2 was also increased in C4-HI tumors as compared to C4-HD tumors (Figure 1A), the role of the RAS-RAF-MEK-ERK1/2 pathway in tumor growth does not seem to be pivotal since PD98059 treatment did not interfere with either C4-HD or C4-HI tumor growth (Figure 1C).

After 12 days of treatment with the inhibitors, animals were euthanized and the tumor samples were excised for protein analysis by western blots. We found a significant reduction (p<0.05) in the levels of p-AKT and p-ERK1/2 in both tumor types as a result of treatment with LY294002 and PD98059, respectively (Figure 1D). This result confirms the effectiveness of these drugs to inhibit their molecular targets. Histological analysis of the tissues shows, as expected, an increase (p<0.01) in the percentage of apoptotic cells in C4-HI tumors treated with LY294002 (Figure 1E). Consistent with the observation that the treatment with PD98059 did not reduce the growth rate of either tumor (Figure 1C) we did not see a significant increase in the apoptosis index in tumors treated with PD98059 by the end of the experiment (Figure 1E).

Finally, we observed that C4-HI tumors, independently of MPA supply, display ductal-like structures (indicated by the black arrows in Figure 1F). These results are consistent with previous studies [7], [40], [42] that show a more glandular-like differentiation pattern in C4-HI than C4-HD tumors. Moreover, treatment with LY294002 causes an increase in this differentiation pattern only in C4-HI tumors (Figure 1F).

### Cancer cells isolated from C4-HD and C4-HI tumors lose differential sensitivity to the inhibition of the PI3K/AKT pathway

**Figure 2:**
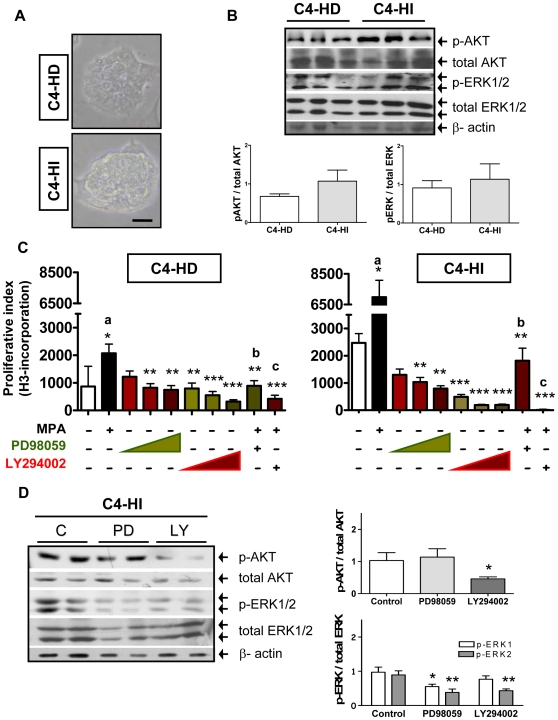
Differential sensitivity to PI3K/AKT pathway regulation is lost in isolated cancer cells. **A**. Phase contrast microscopy of primary cultures isolated from C4-HD and C4-HI tumors showing flattened monolayers on tissue culture plastic. Scale bar: 30 µm. **B**. Top. Western blots showing p-AKT, total AKT, p-ERK1/2, ERK1/2 and β-actin in protein extracts from primary cells cultured for 96 hrs. Three representative samples of a total of six are shown in the gel. Bottom. Quantification of p-AKT/total AKT and p-ERK/total ERK levels, n = 6 for each cell type. **C**. Proliferation assay (^3^H-thymidine incorporation) in primary C4-HD (left) or C4-HI (right) cells incubated during the last 48 hrs with 0.01 µM MPA and 5, 10 and 20 µM PD98059 or LY294002. For the combined treatment, the inhibitor was used at a dose of 10 µM. ^3^H-thymidine was added in the last 18 hrs before harvesting the cells. MPA has a stimulatory effect while PD98059 and LY294002 have an inhibitory effect on MPA-treated and untreated cells. n = 8 for each treatment. ***:p<0.001; **:p<0.01; *: p<0.05 vs. control (untreated cells); different letters indicate significant differences between treatments in the presence of MPA with p<0.01. A representative experiment of a total of three is shown here. **D**. Left. Western blots showing that phosphorylation levels of AKT and ERK1/2 decrease in primary C4-HI cultures incubated for 48 hrs with 10 µM of specific inhibitors. β-actin levels in the blot serve as a loading control. Two representative samples of a total of six are shown (left). Right. Quantification of p-AKT/total AKT and p-ERK/total ERK levels. n = 6 for each group; *p<0.05; **p<0.01 vs. control.

In order to study the mechanisms that lead to the differential activation of AKT in C4-HI and C4-HD tumors, we isolated primary epithelial cells from the tumors and cultured them on plastic tissue culture plates. Under this two-dimensional (2D) condition, both C4-HD and C4-HI epithelial cells grow as clusters that adhere to the plastic (Figure 2A). In contrast to the results obtained with tumors growing *in vivo*, western blot analysis of epithelial cells isolated from C4-HD or C4-HI tumors that were placed on plastic for 96 hours show similar levels of p-AKT and p-ERK1/2 (Figure 2B). Furthermore, analysis of cell proliferation by ^3^H-thymidine uptake revealed that both cell types have a similar responsiveness to MPA or growth factors such as FGF-2 [41], and both display similar sensitivity to the inhibitors PD98059 and LY294002, as shown here (Figure 2C). In both cell types, inhibition of PI3K/AKT and MEK/ERK1/2 signaling interfered with the proliferative effect of 0.01 µM MPA (Figure 2C), suggesting that both pathways are involved in MPA-induced proliferation. Curiously, even though C4-HI tumor cells are MPA-independent *in vivo*, they are MPA-responsive *in vitro*.

As expected, after 10 µM PD98059 and LY294002 treatments, there was a reduction in the levels of p-ERK1/2 (p<0.01) and p-AKT (p<0.05), respectively (Figure 2D) confirming that both inhibitors were able to exert their specific effects. In addition, LY294002 caused a slight decrease in AKT protein levels (Figure 2D). Finally, we also observed a reduction in the levels of p-ERK1/2 in the presence of LY294002 suggesting a functional connection between the PI3K/AKT and MEK/ERK1/2 pathways.

The striking difference between the behavior of tumor cells *in vivo* vs. *in vitro* indicated that, not only hormone regulation, but also the activation of PI3K/AKT and MEK/ERK1/2 signaling pathways, are strongly influenced by the tumor microenvironment and/or host factors. Consistent with this hypothesis are our previous findings demonstrating that C4-HI-derived cancer associated fibroblasts are able to induce PR activation and cell proliferation of epithelial cells more efficiently than C4-HD-derived cancer associated fibroblasts [41]. This discovery indicates that stromal signals are critical in the maintenance of hormone-dependency and can also affect the activation of protein kinases in breast tumors. Naturally, these stromal signals are lost when cancer cells are isolated from the tissue and cultured on tissue culture plastic.

### Differential activation of PI3K/AKT pathway can be maintained in culture when isolated cancer cells preserve their tissue organization

**Figure 3:**
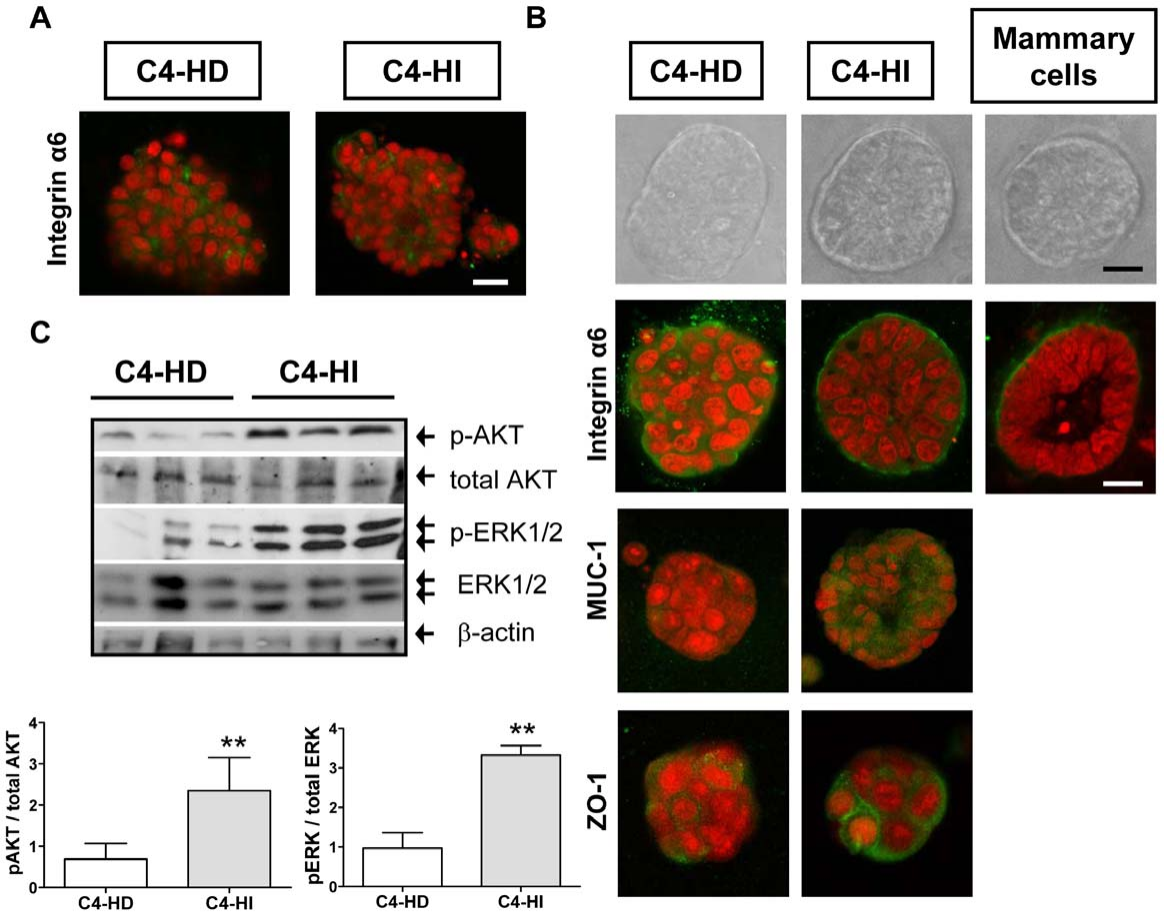
Differential activation of the PI3K/AKT pathway in tumor cells is cell context dependent. Primary cancer cells in 3D cultures “on top” of Matrigel reproduced the architecture typical of the tumors, as well as the differential pattern of protein kinase activation between cells types. **A**. C4-HD and C4-HI primary cells isolated from the tumor and placed on tissue culture plastic for 48 hrs show diffuse and irregular deposition of integrin α6 as assayed by immunofluorescence under confocal microscopy. **B**. Phase contrast microscopy (top) of primary cells placed on top of a thin layer of Matrigel. After 48 hrs, the cells show differences in organization among the three cell types. Confocal images from immunofluorescence studies (bottom) reveal that C4-HI cells (right) form organized clusters with an appropriate continuous basal localization of integrin α6, resembling normal mammary organoids in culture. C4-HI cells exhibit apical localization of MUC-1 on the surface of the cell membrane, lateral localization of tight junction protein ZO-1 and even the presence of a central lumen in some of the clusters. C4-HD cells (left) remain as disorganized clusters, with no clear distribution of any polarity marker tested and no central lumen. Polarity markers were stained green with specific antibodies for immunofluorescence; nuclei were stained red with propidium iodide. Representative clusters of each condition are shown here. Scale bar: 30 µm. **C**. Top. Western blot of protein extracts obtained from primary cultures grown on Matrigel showing that differences on p-AKT and p-ERK1/2 levels between C4-HD and C4-HI tumors is preserved when isolated cells are placed on 3D culture system. β-actin levels in the blot serve as a loading control. Three representative samples of a total of six are shown. Bottom. Quantification of p-AKT/total AKT and p-ERK/total ERK levels. n = 6 for each cell type; **p<0.01 C4-HI vs. C4-HD.

Monolayers of C4-HD and C4-HI primary tumor cells placed on tissue culture plastic lack 3D tissue organization, leading to a loss of normal cell to cell interactions. Under these conditions, immunofluorescence to reveal integrin α6, a protein belonging to a class of extracellular matrix receptors that are normally localized to the basal membrane of polarized cells, showed a disorganized distribution of this protein in epithelial cells derived from both types of tumors, with no polarization pattern (Figure 3A).

In an effort to find an *in vitro* culture system that reproduces the differential phenotype and behavior of C4-HD and C4-HI tumor cells that we observed *in vivo*, we tested the ‘on-top’ assay, in which cells are cultured on top of a thin laminin-rich gel (Matrigel). In these conditions, cells remained as clusters and maintained a 3D structure (Figure 3B). Forty-eight hours after seeding on top of the Matrigel, primary cells derived from C4-HD and C4-HI tumors became enclosed by a rigid structure, and integrin α6 showed basal cell membrane localization by immunofluorescence (Figure 3B). This result suggests that basement membrane components are appropriately deposited. Within this enclosure, most primary C4-HI tumor cells formed polarized and hollow structures, which resemble the lumen present in ductal-like structures found in normal mouse mammary epithelial organoids placed on Matrigel (Figure 3B right). Furthermore, C4-HI cells placed on Matrigel exhibit apical localization of MUC-1 and lateroapical localization of ZO-1 (Figure 3B), a central regulator of tight junction formation. In contrast, most C4-HD tumor cells placed on Matrigel form clusters that are much less polarized, with lower levels of integrin α6, MUC-1 and ZO-1 signal (Figure 3B), and hollow tissue structures are rarely seen.

Moreover, this culture system is reminiscent of the differences in tissue organization observed between C4-HD and C4-HI tumor variants, where C4-HI tumors growing in the absence or presence of MPA show a high degree of differentiation with a ductal-like organization of epithelial cells, while C4-HD tumors are much less differentiated (Figure 1F).

Under these culture conditions, western blots of C4-HI cells showed higher levels (p<0.01) of p-AKT and p-ERK1/2 as compared to C4-HD cells (Figure 3C), resembling the *in vivo* results (Figure 1A,B,D). In conclusion, *in vitro* 3D results reproduced *in vivo* results and revealed that the differences between tumor variants in the activation level of protein kinases could be determined by a particular cell context.

### Differential sensitivity to the PI3K/AKT pathway between tumor cell types is restored under conditions that allow correct tissue organization

**Figure 4:**
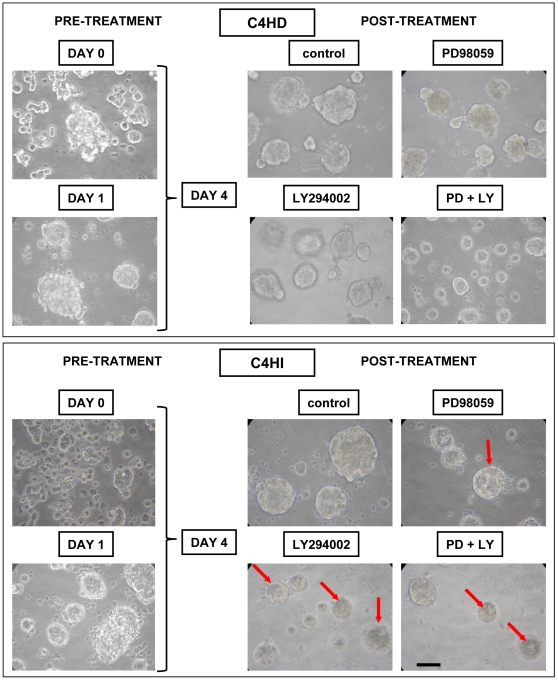
Higher sensitivity of C4-HI tumors to inhibition of PI3K/AKT pathway is restored in 3D Matrigel. The 3D Matrigel culture system reproduces the *in vivo* differential sensitivity of primary cancer cells to PI3K and MEK inhibitors. Phase contrast microscopy showing the progression of C4-HD (top) and C4-HI (bottom) cultures in the presence of 10 µM PD98059, LY294002, or vehicle as control. Day 0 referred to the day of the isolation from the tumors. Primary cultures were maintained for 96 hrs on Matrigel, and the inhibitors were added into the culture medium for the last 48 hrs. During that time, under control conditions the clusters became bigger, due to proliferation and superposition of neighboring clusters. PD98059 causes a small reduction in the size of C4-HI clusters, but the effect of LY294002 is more dramatic on C4-HI than C4-HD cells. A higher number of structures with central lumen (indicated with red arrows) were noticeable in LY294002-treated C4-HI cells. Simultaneous treatment with the two inhibitors equally reduced the size of C4-HD and C4-HI clusters. Scale bar: 30 µm. The same progression was observed in three independent experiments.

**Figure 5:**
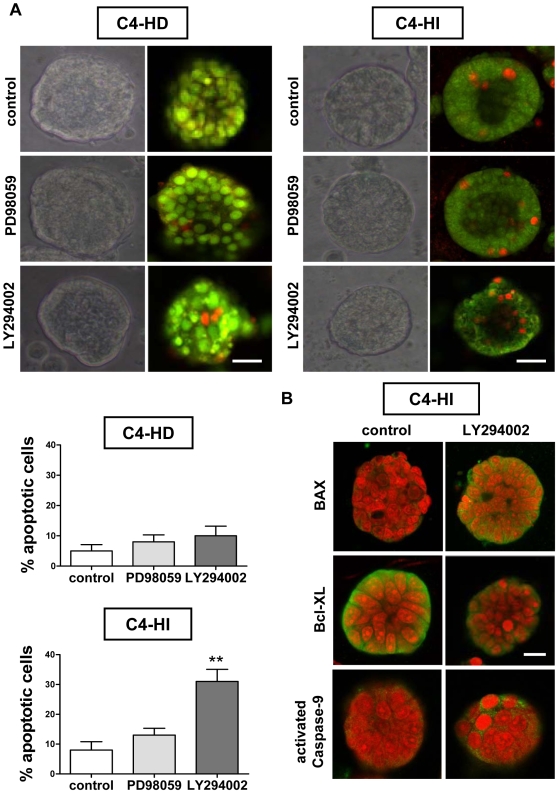
Cell death induced by LY294002 in C4-HI cancer cells involves intrinsic BAX/mitochondrial/caspase-9 apoptotic pathway. The 3D Matrigel culture system of primary C4-HI cancer cells reproduces the increased pro-apoptotic effect of the PI3K inhibitor observed *in vivo*. **A**. Top. Phase contrast microscopy showing a representative C4-HD (left) and C4-HI (right) cell cluster cultured for 96 hrs on Matrigel and treated for the last 48 hrs with 10 µM PD98059, LY294002, or vehicle as control. Confocal images from a fluorescence microscope of acridine orange/ethidium bromide (AO/EB) staining was used to discriminate live from apoptotic cells. AO fluoresces green in live cells and EB fluoresces orange/red when intercalated with DNA in dead cells. Most C4-HI cell clusters on Matrigel exhibit a central lumen. In contrast, no C4-HD cell clusters possess a central lumen. A higher number of apoptotic cells in and around the central lumen of LY294002-treated C4-HI cells was also noted. Scale bar: 30 µm. Bottom. Quantification of the percentage of apoptotic cells per cluster, of four independent experiments with ten clusters in each. Data corresponds to the mean +/− SEM. LY294002 induces cell death in C4-HI cells; **:p<0.01 vs. control. **B**. Confocal images showing higher BAX and activated caspase-9 staining, and lower Bcl-XL staining in C4-HI cells treated with 10 µM LY294002. Nuclei were stained red with propidium iodide. Scale bar: 30 µm.

We then explored the sensitivity of C4-HD and C4-HI cells growing for 96 hrs on Matrigel to PD98059 and LY294002 treatment. Analysis of phase contrast microscopy images revealed critical differences between the two cell types to kinase inhibitor treatment. Similar to what we found *in vivo* (Figure 1E), the PI3K inhibitor reduced cell survival (determined by cluster size) in C4-HI cells significantly more than in C4-HD cells (Figure 4). Furthermore, a small effect was observed using the MEK inhibitor in C4-HI cells. The simultaneous treatment with both inhibitors was remarkably effective both on C4-HD and C4-HI cells in reducing the size of the clusters. Moreover, treatment for 48 hrs with 10 µM LY294002 increased central lumen formation (indicated with red arrows in Figure 4) in C4-HI clusters. To evaluate if there is a selective effect of LY294002 in inducing cell death in C4-HI cells, we used the acridine orange/ethidium bromide (AO/EB) dye incorporation assay. By this technique, apoptotic cells are visualized by their red fluorescence whereas living cells fluoresce green. An analysis of phase contrast microscopy followed by confocal images from a fluorescence microscope of AO/EB staining demonstrated that C4-HD and C4-HI cell clusters were differentially sensitive to protein kinase inhibitors. After 48 hrs of LY294002 treatment, a significant increase (p<0.01) in the number of apoptotic C4-HI but not C4-HD cells was observed. In contrast, PD98059 did not significantly increase the percentage of C4-HI or C4-HD apoptotic cells (Figure 5A). Taken together, these data suggest that C4-HD clusters do not have lumen because of their failure to undergo cavitations via the apoptosis of centrally localized cells (Figure 5A).

To determine the mechanisms by which AKT selectively regulates the survival of C4-HI cells, we measured the levels of pro and anti-apoptotic molecules by immunofluorescence. We found that after treating the cells for 48 hrs with LY294002, there was a decrease in the anti-apoptotic protein Bcl-XL, and an increase both in the pro-apoptotic molecule BAX and activated caspase-9 (Figure 5B).

In conclusion, our results indicate that a major difference between C4-HD and C4-HI cells is the relevant role of the PI3K/AKT pathway in the regulation of cell survival in C4-HI cells and that the activity of this pathway requires an appropriate 3D cell context.

### The activation of AKT is involved in the regulation of ERα levels

**Figure 6:**
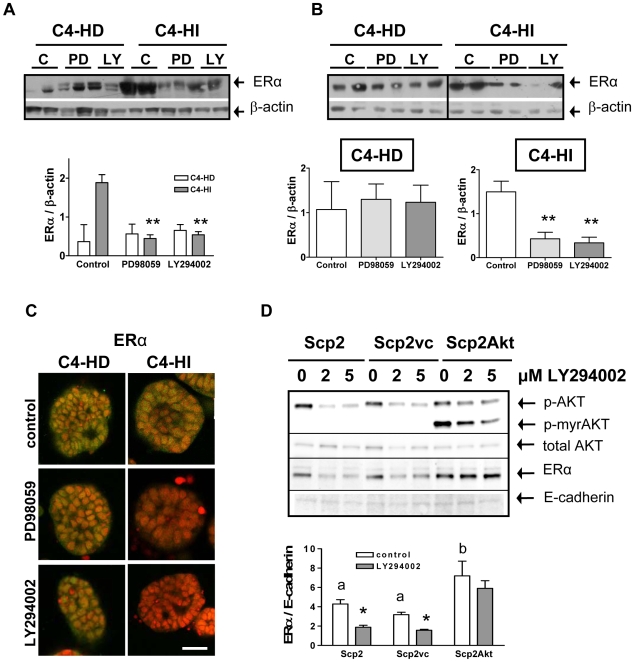
AKT regulates ERα protein expression. **A**. ERα levels determined by western blot are reduced in C4-HI tumors treated with PD98059 or LY294002 administrated as indicated in Figure 1. However, ERα levels are more variable between tumors and not regulated by either inhibitor in C4-HD tumor samples. Top. Two representative samples of a total of six are shown in the gel. Bottom. Quantification of ERα relative to β-actin (used as a loading control) levels. n = 6 for each treatment; **:p<0.01 vs. control. **B**. A similar pattern of regulation of ERα protein is observed in primary cells cultured on top of Matrigel and incubated for 48 hrs with 10 µM of either of the inhibitors. Top. Two representative samples of a total of six of each group are shown in two independent gels. Bottom. Quantificaton of ERα relative to β-actin in each cell type. n = 6 for each treatment; **p<0.01 vs. control. **C**. Representative confocal images showing ERα determined by immunofluoresence (green). Lower levels of the protein are seen in PD98059-treated and in LY294002-treated C4-HI cells growing on Matrigel, whereas similar levels of ERα are seen in C4-HD treated and un-treated cells. Nuclei were stained red with propidium iodide. Scale bar: 30 µm. **D**. Top. A representative sample for each treatment is shown in a western blot. Scp2, a mouse mammary cell line, transfected with a constitutively active form of AKT1, myristoylated AKT1-Δ4-129 (Scp2Akt), displays in the same gel AKT with a typical molecular weight of 59 kDa and the myristoylated deleted variant of AKT1 (p-myrAKT) with a molecular weight of 45 kDa. The antibody used to detect total AKT recognizes only wild type AKT. E-cadherin was used as a loading control for Scp2 cells. Scp2Akt cells exhibit higher levels of ERα than Scp2 cells transfected with the vector control (vc) or Scp2 control cells. In Scp2 and Scp2vc cells, but not in Scp2Akt cells, 2 and 5 µM LY294002 downregulate p-AKT and ERα levels, whereas total AKT levels remains invariable. Bottom. Quantification of ERα relative to E-cadherin levels. n = 4 for each treatment; a,b p<0.05 Scp2Akt vs. Scp2 and Scp2vc; *p<0.05 LY294002 vs. control.

In order to find other mechanisms responsible for the difference in growth between C4-HD and C4-HI tumors, we investigated wether the PI3K/AKT and ERK1/2 pathways regulated the levels of ERα. Inhibition of either pathway significantly (p<0.01) reduced the expression levels of ERα in C4-HI tumors but not in C4-HD tumors as assessed by western blot (Figure 6A). This result, together with our finding that inhibition of p-ERK by PD98059 did not reduce tumor growth rate (Figure 1C), suggest that at least in C4-HI cells, cell proliferation and cell survival are not determined exclusively by ERα levels.

We cultured pure C4-HD and C4-HI primary cells on plastic and then treated them with PD98059 and LY294002. In contrast to the above results, both cell types responded similarly to the inhibitors with a decrease in ERα expression (not shown). Therefore, we decided to grow the cells on Matrigel. When tumor cells were placed on Matrigel, we observed that C4-HI cells exhibited a higher sensitivity, in terms of ERα expression levels, to 10 µM LY294002 and PD98059, than C4-HD cells. ERα levels decreased in C4-HI cells treated with any of the inhibitors for 48 hrs (p<0.01), while ERα levels remained unaltered in C4-HD cells, as determined by western blot (Figure 6B). Immunofluorescence analysis confirmed the results observed by western blot, showing decreased signal for ERα after C4-HI, but not C4-HD cells growing on Matrigel, were treated with the kinase inhibitors (Figure 6C).

Finally, in order to demonstrate that there is a direct relationship between AKT activation and ERα regulation, we transfected Scp2, a non-tumorigenic mouse mammary cell line, with a constitutively active form of AKT1, myristoylated AKT1-Δ4-129 (Scp2Akt). Western blot analysis of these cells revealed a band of 59 kDa corresponding to phospho-Ser473 wild type AKT and a smaller band of 45 kDa corresponding to myristoylated phospho-Ser473 AKT1 (Figure 6D). In Scp2Akt cells ERα expression is increased (p<0.05) in comparison to untransfected Scp2 cells and Scp2 cells transfected with the control vector, Scp2vc (Figure 6D), confirming that ERα expression can be directly regulated by AKT. As expected, 2 and 5 µM LY294002 reduced p-AKT and ERα (p<0.05) levels in Scp2 and Scp2vc cells. Furthermore, the inhibitory effect of LY294002 was smaller in Scp2Akt cells, since constitutively active AKT does not require the activity of PI3K to move to the plasma membrane. This result confirms that the regulatory effect of PI3K occurs through AKT. It is important to mention that the antibody used to detect total AKT recognizes amino acids 71–184 overlapping with the deletion fragment in the myristoylated AKT1, and for that reason the only band observed corresponds to the endogenous, wild type AKT (Figure 6D). E-cadherin protein was used as a loading control for Scp2 cells as previously described [43].

These results indicate that protein kinase signaling can regulate tumor growth by regulating steroid receptor availability in cancer cells, which could shape the response of the tumor to endocrine therapy.

### Differential sensitivity to steroid receptor inhibitors by C4-HD tumor cells

**Figure 7:**
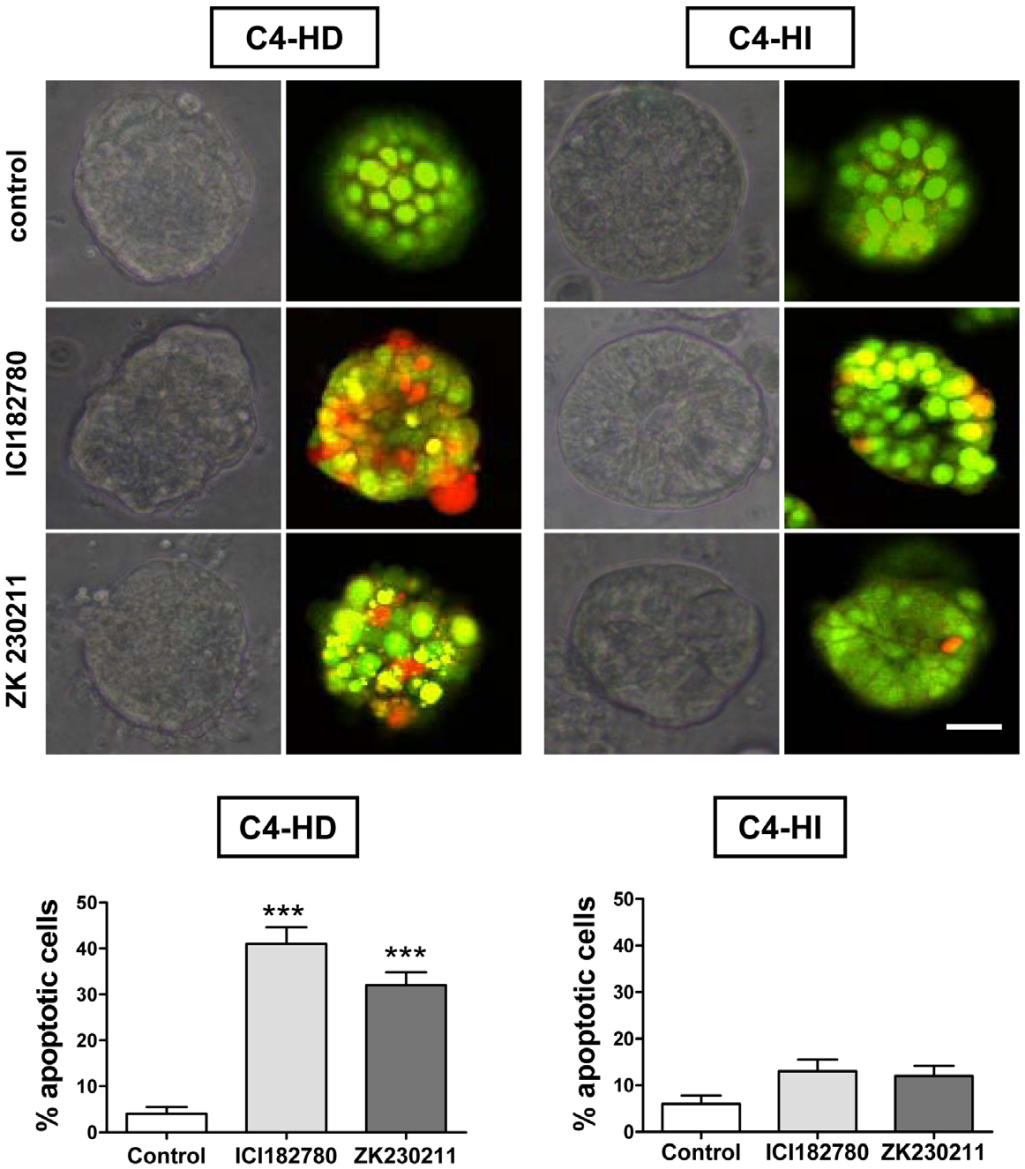
C4-HD tumor cells are more sensitive to steroid receptor inhibitors. Apoptosis induced by inhibitors of ER (ICI182780) and PR (ZK230211) is higher in C4-HD than C4-HI cells. Top: Phase contrast microscopy showing representative C4-HD (left) and C4-HI (right) cell clusters in the presence of 1 µM ICI182780, 0.01 µM ZK230211 or vehicle as control. Confocal images from a fluorescence microscope of AO/EB staining show a higher number of apoptotic cells (red cells) in C4-HD cultures treated with ICI182780 or ZK230211 than in C4-HD untreated cultures. C4-HI cultures exhibit no change in cell death after endocrine treatments. Bottom. Quantification of the percentage of apoptotic C4-HD (left) and C4-HI (right) cells per cluster, of four independent experiments with ten clusters in each. Data corresponds to the mean +/− SEM. ***:p<0.001 vs. control. Scale bar: 30 µm.

We then used the Matrigel culture system to compare the effects of other inhibitors in this model that could be differentially effective in inhibiting C4-HD tumor growth. We tried two well-known steroid receptor inhibitors that are already in preclinical use and are known to be effective in MPA-induced mammary tumors, such as ICI182780, an ER antagonist, and ZK230211, a PR antagonist. Using the AO/EB dye incorporation assay, we found a higher number of apoptotic cells (visualized by red fluorescence) after 48 hrs of treatment with 1 µM ICI182780 or 0.01 µM ZK230211 only in C4-HD tumor cells (p<0.001) (Figure 7, photographs and bar graphs). Moreover, the percentage of apoptotic C4-HI cells did not significantly increase in the presence of any of the steroid receptor inhibitors tested (Figure 7, right bar graph).

These results support the idea that a culture system using Matrigel efficiently maintains *in vitro* the differential cellular responses observed *in vivo* to specific inhibitors that target signaling pathways at different levels. Then, this culture system could be a tool used to find selective antitumor agents against individual tumor types.

### Reconstitution of tissue organization in culture is not sufficient to prevent loss of endocrine resistance of isolated C4-HIR tumor cells

**Figure 8:**
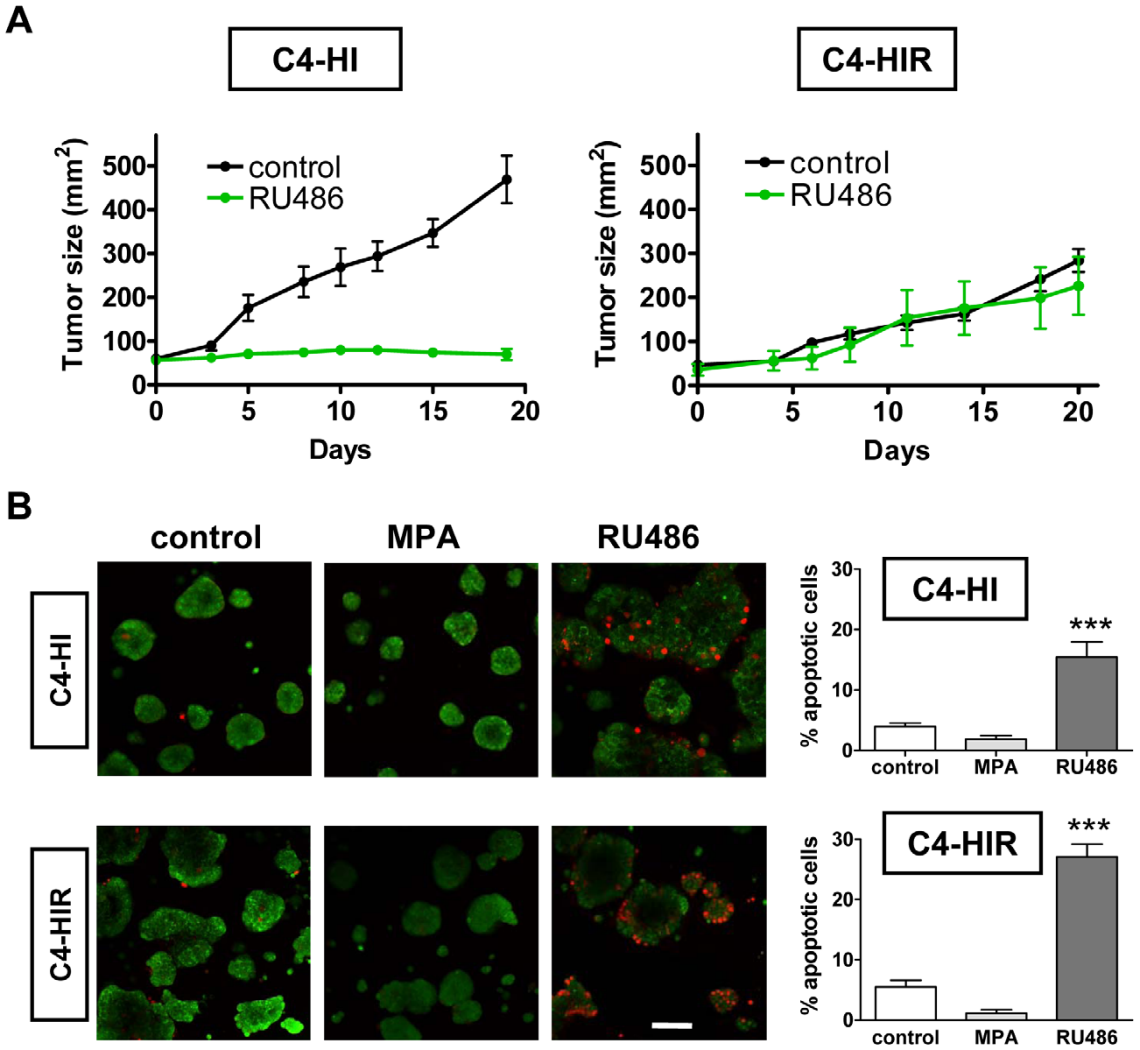
Loss of endocrine resistance in isolated C4-HIR tumor cells is not prevented by 3D Matrigel. Isolated C4-HI and C4-HIR tumor cells are sensitive to cell death in response to RU486 when cultured on plastic, and the 3D Matrigel culture system is not sufficient to revert this phenotype. **A**. C4-HI and C4-HIR tumors were transplanted in syngeneic mice and measured every 2 days (length and width). RU486 was inoculated s.c. at a dose of 12 mg/kg/day once the tumors were 60 mm^2^. C4-HI tumors (left) regress following treatment with antiprogestin, while C4-HIR tumors (right) are unresponsive. **B**. Fluorescent confocal images of AO/EB staining show similar number of apoptotic cells (red cells) after 48 hrs of treatment with 0.01 µM MPA and a higher number of apoptotic cells with 0.01 µM RU486 treatment. Right. Quantification of the percentage of apoptotic C4-HI (upper) and C4-HIR (lower) cells per cluster, of three independent experiments with ten clusters in each. Data corresponds to the mean +/− SEM; ***:p<0.001 vs. control. Scale bar: 100 µm.

Finally, we evaluated whether endocrine resistance of C4-HIR tumors can be reproduced in culture using Matrigel as a substratum. As previously reported [7] and reproduced here (Figure 8A), C4-HI tumors regress after antiprogestin treatment (ZK230211 or RU486). This is in contrast to C4-HIR tumors, which continue growing following the same treatment. However, when primary cells were isolated from each tumor and placed on plastic, both cell types were sensitive to RU486 (unpublished data). Furthermore, this loss of endocrine resistance of C4-HIR tumor cells could not be prevented by culturing the cells on Matrigel. After 48 hrs of 0.01 µM RU486 treatment, both C4-HI and C4-HIR tumor cells were equally sensitive to the antiprogestin, showing similar increase (p<0.001) in the percentages of apoptotic cells (visualized by red fluorescence) when assayed by AO/EB dye uptake (Figure 8B). Under the same conditions, it was noticeable that treatment with 0.01 µM MPA for 48 hrs did not significantly affect basal cell death in both C4-HI and C4-HIR cultures (Figure 8B). It is important to mention that C4-HIR cells remained more disorganized than C4-HI cells on Matrigel (Figure 8B, photographs).

These results indicate that all of the phenomena involved in differential tumor sensitivity to antitumor agents can not be reproduced using Matrigel as a culture system. In the case of endocrine resistance of C4-HIR tumors, other *in vivo* factors might be required to maintain this tumor phenotype.

## Discussion

In this work, we have combined the advantages of using an experimental mouse model that spans the different stages of endocrine responsiveness and mimics critical events in the most frequent type of breast cancer in women [39] with the 3D Matrigel culture system that mimics tissue architecture *in vitro*. Under these conditions, we were able to reproduce *in vitro* many of the *in vivo* behaviors of C4-HD and C4-HI tumors. The ability to do experiments in culture allowed us dissecting some of the mechanisms involved in the acquisition of hormone independence.

We found that AKT is highly active in C4-HI but not in C4-HD tumors and that it regulates C4-HI tumor growth (Figure 1) and cell survival (Figure 5). In contrast, ERK1/2, which is also highly active in C4-HI tumors, is not relevant for tumor growth or cell survival. These results suggest that upregulation of the PI3K/AKT pathway might be a key event in the progression to hormone independence. LY294002 has already been used in preclinical studies [44], [45] and, consisting with the results shown here, its has been shown that its effect in reducing cell survival and tumor growth in mouse thyroid cancers is through a decrease in the phosphorylation of BAD and an increase in proapoptotic caspase 3 [45]. On the other hand, C4-HD tumor cells are more sensitive to steroid receptor antagonists such as ICI182780 and ZK230211 (Figure 7), indicating that in the original tumor variant steroid receptor signaling is prevalent in driving tumor growth and cell survival. Assuming that the signaling pathways that participate in tumor growth and cell survival of each tumor type are indicative of the mechanisms involved in tumor progression, we hypothesize that C4-HI tumors shifted from steroid receptor to the PI3K/AKT signaling pathway dependency. However, our *in vitro* results have shown that only in a 3D Matrigel culture this differential tumor dependency is preserved.

In the future, the 3D Matrigel system will allow us to identify specific regulatory elements missregulated in C4-HI tumors that lead to a hyperactive PI3K/AKT pathway, which might be related to the acquisition of hormone independence. Elucidation of these mechanisms might lead to the development of therapies for preventing and treating hormone-independent breast cancers. Then, an *in vitro* system that preserves *in vivo* differential tumor phenotype, constitutes a prospective tool in finding selective antitumor agents against individual tumor types.

The fact that the dependency of C4-HI tumors on AKT is lost in classic 2D cultures but it is maintained in 3D cultures of nearly pure tumor epithelial cells (compare Figures 2 and 5) indicates that acini-like tissue structure, rather than factors originating in stromal cells, plays a key role on such dependency. Similarly, Zhang and collaborators [46] have shown that estrogen-induced apoptosis of the human ductal breast epithelial tumor cell line T47D:A18/PKCalpha cells is only observed *in vivo* or when cells are grown in Matrigel but not in 2D tissue culture. This is not the case of C4-HIR tumors shown here, which lost resistance to RU486 even in 3D cultures (Figure 8). Of course, not all the phenomena involved in differential tumor sensitivity to antitumor agents can be expected to be reproduced using the Matrigel culture system. For C4-HIR tumors, it is likely that *in vivo* factors, such as carcinoma-associated cells or paracrine signals are required to maintain RU486 resistance. Thus, for C4-HIR tumors, a complementary approach to the 3D culture system might be suitable. For example, Pontiggia et al. used mixed epithelial-stromal cultures (the LM05 cell line) to study estrogen responsiveness and tamoxifen resistance *in vitro* [47]. In their work, the authors revealed that differences between certain tumor variants could be ascribed to the particular stromal cell type of the mix. These findings indicate that breast cancer progression is a very complex phenomenon where alterations of special signaling between particular cellular components could lead to a differential tumor phenotype. This realization led to the recent development of new drugs that instead of targeting the tumor cell, focus on its microenvironment, summarized in references [25], [30], [31].

The PI3K/AKT signaling pathway has also been implicated in altering breast cancer response to multiple therapies [1], [10], [11], [14]. As described in this work, we showed that the inhibitory effect of LY294002 on ERα levels is reduced when constitutively active AKT1 was over-expressed in Scp2Akt cells (Figure 6). Consistent with this result, high levels of AKT activity in myristoylated AKT1 MCF-7 cells confer resistance to the aromatase inhibitor letrozole and to ICI182780 [48]. This resistance is not due to failure of the endocrine agents to inhibit ERα activity; instead, it is characterized by an altered cell cycle and apoptotic response. Beeram et al. [48] found that cotreatment with the mammalian target of rapamycin (mTOR) inhibitor RAD-001 reverses the AKT-mediated resistance and restores responsiveness to antiestrogens. Together, these studies have implications for the design of combination therapies that target alternative pathways and appropriately adapted to particular characteristics of the tumor progression.

In our system, besides its effect on the activation of AKT, LY294002 caused a decrease in ERK activity (Figure 2), suggesting a functional relationship between the two kinases. Furthermore, inhibition of the two pathways by targeting MEK and PI3K produced synergistic effects in inhibiting cell survival (Figure 4), highlighting the interconnectivity of oncogenic signal transduction circuits. The correlation between ERK and PI3K/AKT signaling has been reported in breast cancer cells [29], [49], [50]. Furthermore, Weigelt et al. [28] state that during the acquisition of resistance to targeted therapies, breast cancer cells are able to rapidly adapt to different environments and signaling cues by switching between alternative pathways, specifically PI3K/AKT and RAS-MEK-ERK, that in turn regulate proliferation and cell survival.

In this work, we also found a slight decrease in the protein levels of AKT in response to LY294002 in C4-HI tumor cells (Figure 2) but not in non-malignant Scp2 cells (Figure 6). This effect is in accordance with a study that shows that treatment of aggressive breast cancer cells with β galactoside binding protein (βGBP) cytokine, another functional inhibitor of PI3K, induces apoptosis through a reduction of AKT mRNA levels [50]. Furthermore, our results indicate that LY294002 causes inhibition of tumor growth (Figure 1) and increase in lumen formation in C4-HI cancer cells through an intrinsic BAX/mitochondrial/activated caspase-9 apoptotic mechanism (Figure 5). This is in agreement with other studies that show that suppression of AKT2 expression by shRNA) in MCF-10A cells [51] or mouse mammary epithelial cells derived from Akt1−/− mice [52] restored lumen formation, polarity and luminal apoptosis, with intense activated caspase-3 staining in the presumptive luminal space in 3D Matrigel cultures.

We have previously shown that when C4-HI tumors are exposed to estrogens they regress, and this phenomenon correlates with a down regulation of ERα levels in the epithelial compartment [53]. During tumor regression, there is a reduction in proliferative and antiapoptotic molecules such as cyclin D1 and Bcl-XL, respectively; and an increase in BAX release, leading to the activation of the intrinsic apoptotic mechanism of caspase 9. Finally, reduced ERα levels correlates with an increase in stromal laminin-1 redistribution with a concomitant increase in integrin α6, which contributes to enhance tumor regression by differentiation [53]. In the light of the experiments shown here where LY294002 causes ERα down regulation both in C4-HD and C4-HI tumors (Figure 6) but tumor regression, by apoptosis and differentiation, only in C4-HI tumors (Figure 1), we postulate that AKT regulates C4-HI tumor growth, at least in part, by keeping ERα levels. However, reduced levels of ERα are not sufficient to cause tumor regression because inhibition of ERK1/2, which also reduced ERα levels, did not block tumor growth (Figure 1). The finding of other mechanisms involved in tumor regression could help us to increase the efficacy of tumor therapy to interfere with tumor progression in this model.

Two observations from our studies led us to reconsider the commonly held notion that as breast tumors progress from hormone-dependent to hormone-independent, they become less differentiated and more autonomous. The first observation indicates that C4-HI tumors are more differentiated and display more ductal-like structures than the original C4-HD tumors (Figure 1). This difference is not due to the presence of MPA in the C4-HD tumors because the administration of MPA to C4-HI tumors does not interfere with its pattern of differentiation (not shown). We suspect that in C4-HI tumors the PI3K/AKT and steroid receptor pathways converge into a downstream signal that maintains the observed differentiation pattern in C4-HI tumors. In support of the convergence idea, a) we have previously reported that C4-HI-derived cancer associated fibroblasts are able to induce PR activation and cell proliferation of epithelial cells more efficiently than C4-HD-derived cancer associated fibroblasts [41]; b) we have previously determined that blocking steroid receptors *in vivo* causes C4-HI tumor regression by differentiation and cell death [53], and C4-HD tumors regress exclusively by cell death with no particular spatial pattern [40], [42]; and c) we show here that treatment with LY294002 *in vivo* causes tumor differentiation and regression only in C4-HI tumors (Figure 1). The 3D Matrigel system allowed us to localize apoptotic cells in and around the central lumen of C4-HI cell clusters treated with LY294002 (Figure 5), a phenomenon that correlates with tissue differentiation. We will assess the convergence hypothesis further in future studies.

The second observation indicates that C4-HI tumors are more sensitive to PI3K/AKT and ERK regulation of ERα than C4-HD tumors (Figure 6), and they can maintain such regulation when they are grown on Matrigel. In such a culture system, we have shown that C4-HI cells recover tissue polarity and lumen formation (Figure 3). In previous studies, we have demonstrated that SCg6 cells, a malignant mouse mammary cell line derived from non-malignant Scp2 cells, become unresponsive to basement membrane regulation of ERα expression [54]. These data indicate that C4-HI tumors, although highly metastatic in lymph nodes and lungs [55] are differentiated and are responsive to extracellular matrix signals. These findings suggest that C4-HI tumors could be more sensitive to the combination of PI3K, endocrine and integrin modulators to interfere with their growth. Even the progression from C4-HI to C4-HIR tumors could be impeded with such combinatorial treatment. Future studies will be aimed to test this hypothesis in animals.

In conclusion, based on the biomarkers of tumor progression resulting from the studies in 3D cultures of the MPA breast cancer model, it will be possible in the future to design and test multi-targeted treatments involving a combination of selective inhibitors of endocrine response, protein kinases and extracellular matrix signals. Our study contributes to a relevant preclinical model system that is suitable for testing the effectiveness of novel therapies in targeting the whole tumor and not just the epithelial component. Furthermore, the animal model that we used here has the added advantage that it is composed of several tumor types that were independently derived (the one presented in this study is C4). In the future, we can determine if the processes that lead to hormone independency and resistance are general and not a unique event that occurs in this particular type of tumor.

## Materials and Methods

### Animals

Two-month-old virgin female BALB/c mice (IBYME Animal Facility) were used. All animal procedures were approved by the Ethical Committee from the Institute of Experimental Biology and Medicine (IBYME): Dr. Enrique Segura, Dr. Ricardo Calandra, Dr. Claudia Marro, Dr. Alberto Baldi and Dr Carlos Libertum. Animal care and manipulation were in agreement with institutional guidelines and the Guide for the Care and Use of Laboratory Animals [56].

### Tumors

Hormone-dependent C4-HD is a transplantable ductal mammary tumor that is maintained by serial subcutaneous (s.c.) transplantations into medroxyprogesterone acetate (MPA)-treated syngeneic BALB/c female mice. Tumor growth is induced by a s.c. depot of MPA (20 mg) in the contralateral flank of the mice [38]. A hormone-independent tumor variant named C4-HI was derived from a C4-HD tumor that grew in a mouse that had not been treated with MPA. Both C4-HD and C4-HI tumor variants express ER and PR and regress once silastic pellets of antiprogestin RU486 (6 mg/kg) were s.c. implanted in the back of the animals [42].

A group of females carrying C4-HD or C4-HI tumors was inoculated i.p. every other day for 12 days with saline solution (control), PD98059 (3.6 mg/kg) or LY294002 (4 mg/kg). Doses were adapted from the literature [57], [58] and [44], [45], respectively. The tumor size was evaluated every 2 days using a *Vernier* caliper to calculate tumor area (length and width) in mm^2^. Treatments with the inhibitors started once the tumors reached a size of approximately 30 mm^2^.

The generation of tumors with acquired resistance to antiprogestin, C4-HIR, was performed by s.c. administration of RU486 (12 mg/kg/day) to mice carrying C4-HI tumors as described previously [7] and maintained by syngeneic transplantation.

All experiments involving animals were repeated two or three times using at least three mice per group each time, as indicated in each figure.

Tumors smaller than 150 mm^2^ growing in each determined condition were excised after euthanasia of the animals and immediately frozen at −80°C for western blots or formalin-fixed for immunohistochemistry studies.

Paraffin sections were stained with hematoxylin-eosin. Sections were analyzed using a Nikon Eclipse E800 Microscope and images were taken with Nikon DS-U1 with ACT-2U (for Nikon) software.

Neither PD98059 nor LY294002 had a toxic effect after 12 days of treatment, as determined by histological evaluation of kidney, spleen and liver (data not shown).

### Culture media and drugs

DMEM/F12 (Dulbecco's modified Eagle's medium: Ham's F12, 1∶1, without phenol red, Sigma Chemical Company, St. Louis, MO, USA), 100 U/ml penicillin and 100 µg/ml streptomycin with 2% (for on top of Matrigel cultures) or 10% (for on plastic cultures) fetal calf serum (FCS) (Bioser, Buenos Aires, Argentina).

PD98059 and LY294002 were obtained from Calbiochem, La Jolla, CA; RU486 (mifepristone) from Sigma Chemical Company, St. Louis, MO. MPA was kindly provided from Craveri Laboratorios, Buenos Aires, Argentina; ZK230211 was kindly provided by Bayer Schering Pharma AG, Berlin; and ICI182780 (fulvestrant) was kindly provided by AstraZeneca London, United Kingdom.

### Mouse mammary epithelial cells

Primary mammary epithelial organoids were prepared by a procedure described previously [43] using the 4th inguinal mammary glands from nulliparous two-months virgin BALB/c mice. Epithelial organoids were resuspended in 2% FCS DMEM/F12 growth medium on top of Matrigel.

### Scp2 cell line

A functionally normal mouse mammary epithelial cell line, Scp2 [59] was kindly provided by Dr. Mina Bissell (LBNL, Berkeley, CA) and maintained in 2% FCS DMEM/F12 on tissue culture plastic. Scp2 cells were transfected using Lipofectamine 2000 (Invitrogen Corporation) with a pWZL plasmid containing myristoylated AKT1, kindly provided by Dr. Richard Roth (Stanford, CA). This AKT1 variant lacks amino acids 4 to 129 (Δ4–129) and bears a myristoylation signal that causes its constitutive activation [60]. Scp2 transfected with myristoylated AKT1 were named Scp2Akt. Scp2 cells transfected with empty pWZL plasmid were named Scp2vc. The cells were lysed using M-PER mammalian protein extraction reagent (Pierce, Rockford, IL) 48 hrs after transfection, and prepared for western blotting.

### Tumor primary cultures

Epithelial cell clusters were separated by differential sedimentation from C4-HD, C4-HI or C4-HIR tumors as indicated in [38] and plated with 2% or 10% FCS, as indicated above. The cells were maintained with the indicated medium for 48 hrs. Then, the medium was replaced by 0% FCS DMEM/F12 for another 48 hrs. During this period, different drugs were added to the 0% FCS medium, such as 5, 10 or 20 µM PD98059; 5, 10 or 20 µM LY294002, 1 µM ICI182780, 0.01 µM ZK230211, 0.01 µM MPA, and 0.01 µM RU486, or the vehicle (DMSO) as a control.

### Cultures in 3D

For 3D cultures, approximately 10^5^ epithelial cells/ml were seeded on top a reconstituted basement membrane gel (Matrigel®, phenol red free, growth factor reduced Matrix, BD Biosciences-Discovery Labware) according to [21], [43]. The Matrigel coverage was prepared according to the manufacturer's instructions by using 70 µl of Matrigel to cover an 8-well Lab Tek Permanox chamber slide (Nalge Nunc International). For western blot assays 140 µl of Matrigel were used to cover each well of a 12-well plate. After isolation from the tumor, epithelial cells were seeded on top of the Matrigel, in 2% FCS-DMEM/F12 medium. After 48 hrs, the medium was removed, and all the experiments and treatments were carried out in serum free DMEM/F12 medium. The cells were incubated for other 48 hrs in the presence of PD98059, LY294002, ICI182780, ZK230211, MPA, or RU486, as indicated. The volume of Matrigel was used to calculate the final concentration of the compounds. At the end of the treatment, the medium was removed, and the gel containing the cells was gently washed twice with PBS.

### Apoptosis

Apoptosis in the tumor tissue was morphologically determined in paraffin sections previously stained with hematoxylin-eosin. The percentage of apoptosis was calculated as the number of cells undergoing apoptosis over the total number of cells in ten high-power fields.

Cell apoptosis in culture was evaluated by staining the cells on top of the Matrigel for 10 seconds with acridine orange (AO) and ethidium bromide (EB) for discrimination of live from dead cells on the basis of membrane integrity [61], [62]. The final concentration of dye mix was 4 µg/ml AO and 4 µg/ml EB in PBS. AO/EB staining was used to visualize nuclear changes and apoptotic body formation. Live cells fluoresce green (with acridine orange) and dead cells fluoresce orange/red (with ethidium bromide that intercalates with double-stranded DNA). Images were taken using a fluorescence confocal Nikon C1 microscope equipped with excitation and emission filters for acridine orange (488 nm and 520 nm, respectively) and for ethidium bromide (544 nm and 570-LP nm). Percentage of apoptotic cells was calculated as the number of red cells over the total number of cells in each cluster in ten clusters.

### Cell proliferation

A ^3^H-Thymidine uptake assay was performed as previously described [63]. Briefly, in a Corning 96-well microplate, 0.1 ml/well of a cell suspension was seeded directly (with no Matrigel coverage) at a concentration of 10^5^ cells/ml. After attachment (48 hrs), the cells were incubated for another 48 hrs with the experimental solutions to be tested. The cells were incubated with 0.4 µCi of^ 3^H-thymidine (specific activity: 20 Ci/mmol) for the last 18 hrs, trypsinized and harvested in a cell harvester. Filters were counted in a liquid scintillation counter. Assays were performed in octuplicates and the mean and standard deviation were calculated for each solution tested.

### Immunohistochemistry

Formalin-fixed, paraffin-embedded tissues were reacted with the phosphorylated Ser473 AKT (p-AKT) antibody (sc-33437, from Santa Cruz Biotechnology, CA) using the avidin/biotin peroxidase complex technique (Vectastain Elite ABC kit; Vector Laboratories, Burlingame, CA). The reactions were developed with 3-3′diaminobenzidine (DAB) as described [42]. Primary antibody was used at 1∶100 dilution and incubated overnight at 4°C. After immunohistochemistry, the specimens were lightly counterstained with 10% hematoxylin, dehydrated, and mounted.

### Immunofluorescence

Cell clusters seeded on top of Matrigel in chamber slides were washed and fixed in 10% formalin for 20 minutes at room temperature. Fixed clusters were treated with primary antibodies to integrin α6 (555734, from BD Pharmigen, San Diego, CA); MUC-1 (62687) and activated caspase-9 (52298) from Abcam, Cambridge, UK; ZO-1 (61–7300) from Zymed Laboratories, San Francisco, CA; BAX (sc-526), Bcl-XL (sc-634) and ERα (sc-542) from Santa Cruz Biotechnology, CA. The antibodies were dissolved in blocking buffer at appropriate dilution and incubated overnight at 4°C. The corresponding secondary FITC-conjugated antibodies (Vector Laboratories Burlingame, CA) were dissolved at 1∶100 dilution and incubated for 1 hr at room temperature. The nuclei were stained with propidium iodide. Slides were mounted with Vectashield (Vector Laboratories, Burlingame, CA) and analyzed under a Nikon C1 Confocal Microscope using the EZ-C1 2.20 software and a PlanApo 40X/0.95 objective.

### Protein extraction and western blots

Tumors were homogenized and processed to obtain total fractions for western blot as described previously [41]. To prepare cell culture total extracts, the cells were lysed using M-PER mammalian protein extraction reagent (Pierce, Rockford, IL). For protein extraction of primary cells grown on top of Matrigel, the cell clusters were previously removed from the gel, with a gently digestion of the gel using Matrisperse BD Cell Recovery Solution (BD Biosciences - Discovery Labware) according to manufacturer's instructions. Once the clusters were recovered, cell lysis was performed using M-PER reagent. Similar amounts of protein extracts as determined by Lowry were loaded into each lane. Western blot were performed and the membranes were incubated with antibodies specific for ERα (sc-542), ERK (sc-94) and p-ERK (sc-7383) all purchased from Santa Cruz Biotechnology; total AKT (610837) and E-cadherin (610181) from BD Transduction Laboratories; phosphorylated Ser473 AKT (p-AKT) (9271) from Cell Signaling Tech, Danvers, MA; β-actin (clone ACTN05) from Neomarkers, Lab Vision Corp. All primary antibodies were incubated overnight at 4°C at a final concentration that was suggested by manufacturer's instructions.

### Statistical analysis

Western blot band intensity and cell staining were quantified using the Image J software. ANOVA and the Tukey multiple post t test were used to study the differences of means of multiple samples; the Student's t test was used to compare the means of two different groups. Tumor growth curves were studied using regression analysis, and the slopes were compared using ANOVA followed by parallelism analysis. Data analysis was performed using the Graph Prism 4.0 software.
